# Mitchell-Riley Syndrome: A Novel Mutation in RFX6 Gene

**DOI:** 10.1155/2015/937201

**Published:** 2015-12-03

**Authors:** Marta Zegre Amorim, Jayne A. L. Houghton, Sara Carmo, Inês Salva, Ana Pita, Luis Pereira-da-Silva

**Affiliations:** ^1^Genetics Department, Hospital Dona Estefânia, Centro Hospitalar de Lisboa Central, 1169-045 Lisbon, Portugal; ^2^Royal Devon and Exeter Hospital, Exeter, Devon EX2 5DW, UK; ^3^Pediatric Surgery Department, Hospital Dona Estefânia, Centro Hospitalar de Lisboa Central, 1169-045 Lisbon, Portugal; ^4^NICU, Hospital Dona Estefânia, Centro Hospitalar de Lisboa Central, 1169-045 Lisbon, Portugal

## Abstract

A novel RFX6 homozygous missense mutation was identified in an infant with Mitchell-Riley syndrome. The most common features of Mitchell-Riley syndrome were present, including severe neonatal diabetes associated with annular pancreas, intestinal malrotation, gallbladder agenesis, cholestatic disease, chronic diarrhea, and severe intrauterine growth restriction. Perijejunal tissue similar to pancreatic tissue was found in the submucosa, a finding that has not been previously reported in this syndrome. This case associating RFX6 mutation with structural and functional pancreatic abnormalities reinforces the RFX6 gene role in pancreas development and *β*-cell function, adding information to the existent mutation databases.

## 1. Introduction

In the rare Mitchell-Riley syndrome (OMIM #601346) recently described, severe neonatal diabetes associated with hypoplastic or annular pancreas, duodenal or jejunal atresia, intestinal malrotation, gallbladder hypoplasia or agenesis, and cholestatic disease are the most common features described [1, 2].

Homozygosity mapping has identified chromosomal regions linked to Mitchell-Riley syndrome, in which the regulatory factor X 6 (RFX6) gene was identified [3]. The RFX family has 7 members of transcription factors with a highly conserved DNA-binding domain. The RFX3 and RFX6 members are important for *β*-cell formation and function [4]. Mutations in RFX6 are assumed to be the cause of neonatal diabetes in this syndrome, through the production of a defective RFX6 protein [2–5]. Recently, Concepcion et al. [2] reviewed the RFX6 mutations found in the eight patients (seven probands) reported to date with neonatal diabetes and multiple congenital digestive tract anomalies.

Herein, a novel RFX6 mutation is reported in an infant with Mitchell-Riley syndrome.

## 2. Case Report

We report a girl, the second child of first-cousin Gipsy parents. Before the proband, the 28-year-old mother had 3 spontaneous abortions in the first trimester and a child with isolated congenital anorectal malformation.

The ultrasound scanning at 33 weeks of gestation revealed intrauterine growth restriction without umbilical artery flow changes, and a “double bubble” suggestive of duodenal atresia. A normal 46, XX karyotype was observed in fetal cells and the fetal echocardiogram was normal. She was born at 35 weeks of gestation by spontaneous vaginal delivery. At birth she appeared malnourished with weight, length, and head circumference below the 3rd centile, respectively, 1370 g, 44 cm, and 29.5 cm. No dysmorphic features were noted, except for anteriorly placed anus. The first hours after birth were complicated with anemia (hemoglobin 9.8 g/dL), oliguria, and metabolic acidosis, improving with volume expansion and red blood cell transfusion. On day 2 hyperglycemia (415 mg/dL) occurred, requiring continuous insulin infusion along with parenteral nutrition with glucose rates around 5-6 mg/Kg/min. On day 3 the blood insulin level was 0.12 *µ*UI/mL and C peptide was <0.1 ng/mL during an episode of hyperglycemia (312 mg/dL). Glutamic acid decarboxylase (GADA), islet cell (ICA), and islet antigen 2 (IA-2) autoantibodies were negative.

On day 4 laparotomy confirmed duodenal atresia (type II) that was associated with annular pancreas, malrotation, and gallbladder agenesis. Perijejunal tissue in the submucosa was found with macroscopic appearance of ectopic pancreatic tissue; instability of the patient during surgery precluded the biopsy of the tissue for histological confirmation. Duodenoduodenostomy and Ladd's procedures were performed. Preoperative analyses showed bilirubin and liver enzymes within normal values. From D6 cholestasis appeared and progressively worsened reaching levels of conjugated bilirubin of 3.07 mg/dL, gamma-glutamyl transpeptidase of 458 IU/L, with relatively normal alkaline phosphatase 94–149 IU/L and transaminases. Subsequently, cholestasis progressively improved and normalization occurred by two months of age without any specific treatment. On day 7 the abdominal ultrasound showed biliary duct dilation with 3 mm greater axis, slightly tortuous, with diminished caliber of intrahepatic right and left biliary ducts; the cephalic region of pancreas was disproportionately larger than the body with unidentifiable tail.

Enteral nutrition was initiated on day 9; however diarrhea developed when enteral volume of breast milk and/or extensively hydrolyzed formula was increased. Exocrine pancreatic supplementation has been unsuccessful and even with free amino acids formula she maintained diarrhea, limiting enteral feeding progression.

Currently she is seven months old and still has malabsorption with failure to thrive (weight 4865 g). Insulin was administered by continuous infusion up to age of five months and subsequently by insulin pump. Complementary feeding was initiated at the age of six months, but she is still dependent on free amino acids formula and parenteral nutrition which provides approximately 60% of daily energy.

The proband and her parents underwent genetic analysis of RFX6 gene. Genomic DNA was extracted from peripheral leukocytes using standard procedures and the coding region and intron/exon boundaries of the RFX6 gene were amplified by PCR (primers available on request). Amplicons were sequenced using the Big Dye Terminator Cycler Sequencing Kit v3.1 (Applied Biosystems, Warrington, UK) according to manufacturer's instructions and reactions were analysed on an ABI 3730 Capillary sequencer (Applied Biosystems, Warrington, UK). Sequences were compared with the reference sequences (NM_173560.3) using Mutation Surveyor v3.24 software (SoftGenetics, State College, PA). A homozygous missense mutation was identified on exon 4, c.541C>T, p.R181W in the proband. Both parents are heterozygous.

## 3. Discussion

In permanent neonatal diabetes mellitus a genetic cause can be identified in around half the cases and the altered gene expression usually affects pancreas development, *β*-cell mass, or *β*-cell function [4]. The most frequent gene with identified mutations is KCNJ11 and to a lesser extent GCK, ABCC8, and HNF1*β*. Most patients with neonatal diabetes do not have other congenital abnormalities [6]. The association of the particular phenotype of Martinez-Frias syndrome with a mutation on the RFX6 gene and neonatal diabetes has been called Mitchell-Riley syndrome, and it was suggested that both syndromes represent a symptom continuum or an RFX6 malformation complex [7].

The most common features described in Mitchell-Riley syndrome were present in the reported case, including severe neonatal diabetes associated with annular pancreas, intestinal malrotation, gallbladder agenesis, abnormal biliary tract, cholestatic disease, chronic diarrhea, intrauterine growth restriction, and consanguinity [1, 2, 4]. Some less common features have also been reported [1, 2]. Similarly to our case, anemia at birth requiring prompt red blood cell transfusion has been previously described [1, 5, 8]. Precocious anemia may have been due to fetomaternal transfusion, but this was not investigated. A novel feature found in our case was the perijejunal tissue similar to pancreatic tissue in the submucosa. Unfortunately, instability of the patient during surgery precluded the biopsy for histological confirmation. Two theories have been suggested for heterotopic pancreatic tissue: during embryological development buds of embryonic tissue penetrate into the wall of the growing gut separating from the main pancreas; alternatively, inappropriate expression of pluripotent embryonic mesenchymal tissue of the gastrointestinal tract may lead to pancreatic metaplasia [9–11].

All the seven probands previously reported with Mitchell-Riley syndrome presented RFX6 mutations [2]. To the best of our knowledge this is the eighth proband with Mitchell-Riley syndrome and RFX6 mutation, in whom the novel p.R181W mutation was found. The arginine residue at codon 181, located in the DNA binding domain, is highly conserved across species and this change affects protein function. This mutation was not listed before and is likely pathogenic.

A different mutation at the same residue has already been reported in patients with Mitchell-Riley syndrome (p.R181Q) [4].

In Mitchell-Riley syndrome accurate genetic counselling is inseparable from molecular diagnosis, offering better reproductive options and prenatal diagnosis to the couple and genetic counselling to family members at risk.

The present report reinforces that severe neonatal diabetes associated with RFX6 gene mutation constitutes a distinct phenotype presently described as Mitchell-Riley syndrome. This strongly suggests that RFX6 has a specific role in pancreas development and *β*-cell function. The novel mutation herein described may contribute to clarifying the reported genetic heterogeneity and adds valuable information to the existent mutation databases.
